# Exploring Non-orthosteric Interactions with a Series of Potent and Selective A_3_ Antagonists

**DOI:** 10.1021/acsmedchemlett.1c00598

**Published:** 2022-01-10

**Authors:** Darío Miranda-Pastoriza, Rodrigo Bernárdez, Jhonny Azuaje, Rubén Prieto-Díaz, Maria Majellaro, Ashish V. Tamhankar, Lucien Koenekoop, Alejandro González, Claudia Gioé-Gallo, Ana Mallo-Abreu, José Brea, M. Isabel Loza, Aitor García-Rey, Xerardo García-Mera, Hugo Gutiérrez-de-Terán, Eddy Sotelo

**Affiliations:** ^†^Centro Singular de Investigación en Química Biolóxica e Materiais Moleculares (CIQUS) and ^‡^Departamento de Química Orgánica, Universidade de Santiago de Compostela, 15782 Santiago de Compostela, Spain; § Department of Cell and Molecular Biology, SciLifeLab, Uppsala University, Uppsala SE-75124, Sweden; ∥ Centro Singular de Investigación en Medicina Molecular y Enfermedades Crónicas (CIMUS). Universidade de Santiago de Compostela, 15782 Santiago de Compostela, Spain

**Keywords:** A_3_ Adenosine receptors, Adenosine antagonists, Pyrimidines, Ugi reaction, Multicomponent reactions

## Abstract

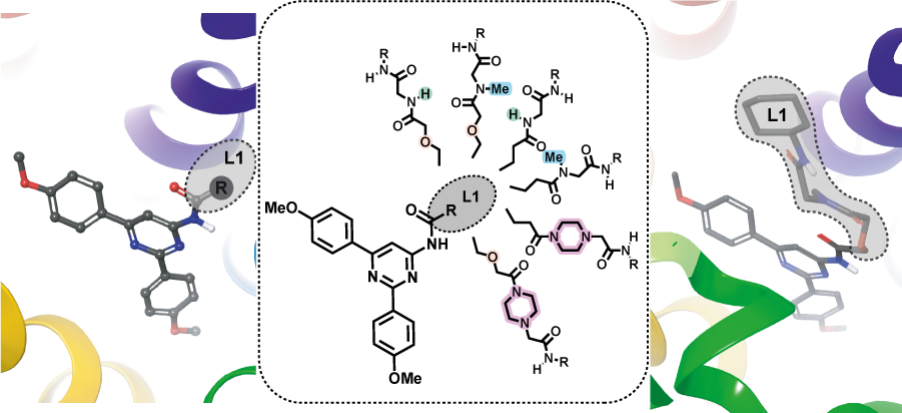

A library of potent and highly A_3_AR selective pyrimidine-based compounds was designed to explore non-orthosteric interactions within this receptor. Starting from a prototypical orthosteric A_3_AR antagonist (ISVY130), the structure-based design explored functionalized residues at the exocyclic amide L1 region and aimed to provide additional interactions outside the A_3_AR orthosteric site. The novel ligands were assembled through an efficient and succinct synthetic approach, resulting in compounds that retain the A_3_AR potent and selective profile while improving the solubility of the original scaffold. The experimentally demonstrated tolerability of the L1 region to structural functionalization was further assessed by molecular dynamics simulations, giving hints of the non-orthosteric interactions explored by these series. The results pave the way to explore newly functionalized A_3_AR ligands, including covalent drugs and molecular probes for diagnostic and delivery purposes.

The endogenous nucleoside adenosine (Ado) is essential for the correct operation of all the mammalian cells.^1,2^ Most of Ado’s actions are triggered by the activation of four membrane receptors, namely, adenosine receptors or ARs (e.g., A_1_AR, A_2A_AR, A_2B_AR, and A_3_AR).^2,3^ From a pathological point of view, extracellular levels of Ado result in two well-defined and opposed effects.^4−6^ While in some cases it is shown to impede the progression of the disease, in others the overproduction of Ado has a protective and stimulant effect that facilitates the progression of the pathology. Hence, regulation of the adenosinergic signaling pathways is emerging as a highly versatile approach addressing clinical challenges in a variety of therapeutic fields.^7,8^

Adenosine A3 receptor (A3AR) is the most recent AR subtype to be characterized,^9^ albeit not yet at the structural level. Expressed in heart, brain, lung, colon, and immune cells, activation of A_3_AR inhibits adenylate cyclase, increases phosphatidylinositol phospholipase C and D activity, and elevates intracellular Ca^2+^ and inositol 1,4,5-trisphosphate levels. A_3_AR is heavily implicated in a variety of cardiovascular and neurological disorders^10^ but is also overexpressed in several cancer cells,^5^ making them a possible biomarker for cancer diagnosis, prognosis, and therapeutic monitoring. Despite being recognized as an attractive target to treat several pathologies (e.g., cancer, rheumatoid arthritis, or glaucoma), A_3_AR remains an enigmatic and controversial receptor.^11,12^ Its contradictory signaling and dual behavior in various pathological conditions highlight that the molecular basis of A_3_AR function remains elusive.^10,11^ Perhaps for these reasons only few A_3_AR ligands reached advanced preclinical characterization or clinical trials.^13,14^ As far as we know, Palobiofarma ligands PBF-677 and PFB-1650 (structures not disclosed) are the only A_3_AR antagonists that entered clinical studies (for ulcerative colitis and psoriasis, respectively).^15^ Furthermore, A_3_AR biased modulator FM101 is also in clinical trials for glaucoma and hepatitis.^16,17^ Hence, there is a growing demand of A_3_AR modulators and pharmacological tools enabling researchers to better define its function in pathologic and physiologic settings and thus unequivocally validate its therapeutic potential.

Our laboratories have been recently focused on the development of potent and subtype-selective A_3_AR antagonists.^18−20^ Our innovative approach combines concise and efficient synthetic methodologies with structure-based computer-aided design, allowing the identification of interesting scaffolds, including 4-amido-2,6-diarylpyrimidines^18^ (Figure 1), which were demonstrated to be superior to their regiosiomers (e.g., 2-amido-4,6-diarylpyrimidines). The binding model generated for this scaffold involved a double hydrogen bond with Asn250^6.55^ and π–π stacking with Phe168^EL2^,^18,19^ conserved among all ARs, while the L2 and L3 fragments were optimally accommodated within transmembrane (TM) regions TM5-TM3 and TM2-TM7, respectively (Figure 1).^19^ The modeling allowed us to explain the initial structure–activity relationship (SAR) within these series and the marked selectivity for the A_3_AR, resulting from specific interactions in the pocket surrounding the L2 substituent, but most importantly it drove the optimization of the substitution patterns for the aryl fragments^18,19^ as well as the superior affinity of pyrimidine versus pyridine scaffolds (Figure 1).^20^

**Figure 1:**
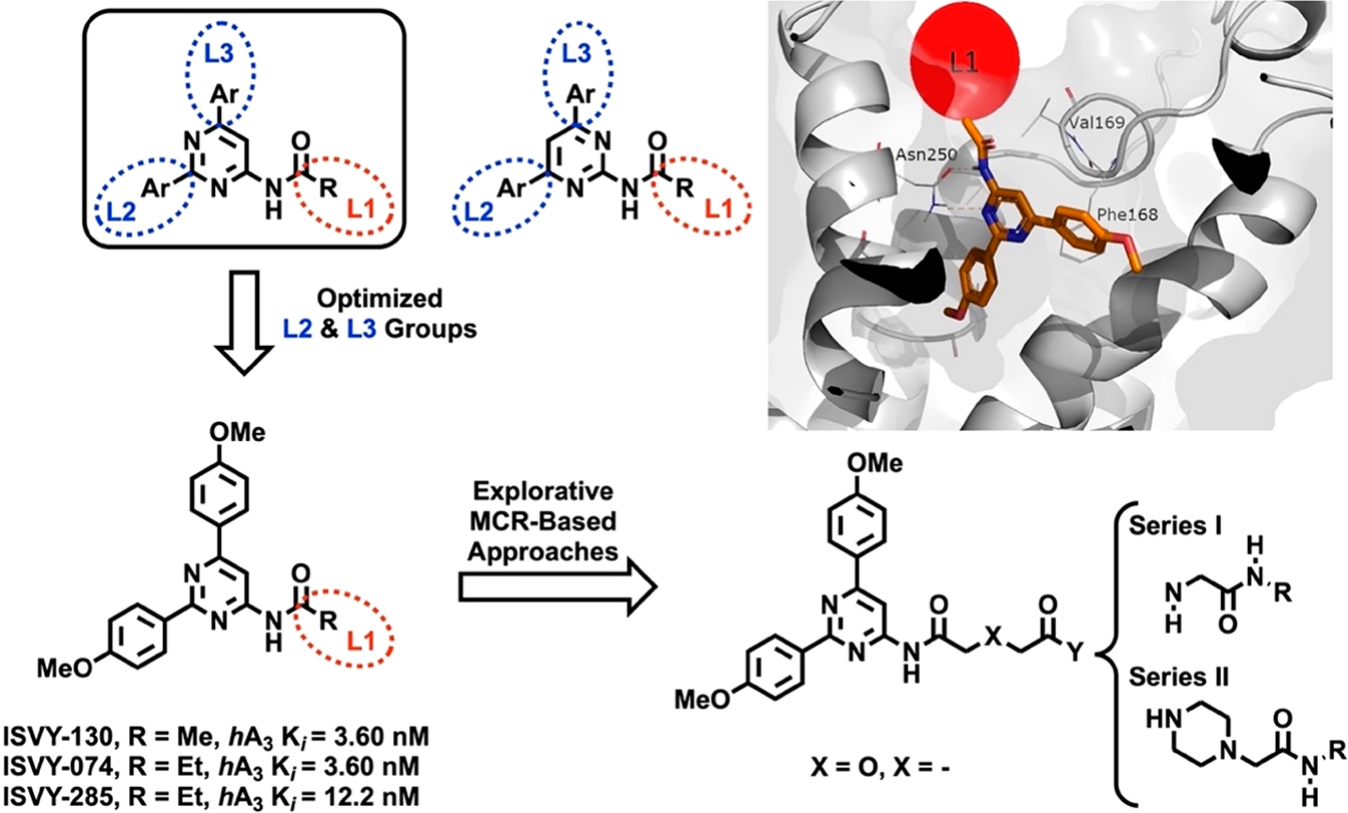
Model A_3_AR antagonists, design strategy, and structure of herein explored ligands.

In the present study, we further explore a prototypical hit compound generated in those studies (Figure 1) by incorporating functionalized residues at the L1 region, designed to provide additional interactions out the A_3_AR orthosteric site. The novel ligands retain the A_3_AR potent and selective profile while improving the solubility, paving the way for the development of newly functionalized A_3_AR ligands, including covalent drugs and molecular probes for diagnostic and delivery purposes.

The synthesis of targeted structures required the structural derivatization of the 4-amino-2,6-di(4-methoxyphenyl)pyrimidine (**1**). Treatment of **1** with either succinic (**2a**) or glycolic anhydride (**2b**) afforded the corresponding carboxylic acids **2a**,**b** (Scheme 1), which contain the pharmacophore while lightly differing in length and the presence of the heteroatom. Carboxylic acids (**2a**,**b**) were employed as reactive precursors for the assembly of exploratory series **I** and **II** (Scheme 1, compounds **7**–**10**) using two convergent and highly reliable Ugi-based transformations (Scheme 1). Equimolar amounts of **2a**,**b**, formaldehyde (**3**), ammonia or methylamine (**4a**,**b**), and three isocyanides (**5a**–**c**) were treated under the Ugi reaction conditions (U-4CR), in methanol at room temperature (48 h), affording the targeted adducts **7** and **8** (series **I**) with satisfactory yields. In a similar fashion but substituting the primary amines (**4a**,**b**) with piperazine, the piperazinyl derivatives **9** and **10** (series **II**) were obtained. All ligands obtained were isolated and subsequently purified by column chromatography, rendering the target structures in moderate to satisfactory yields (45–78%). A comprehensive account of the synthesis, spectroscopic and analytical data for reported ligands, as well as the HPLC traces of representative ligands herein described are provided in the Supporting Information.

**Scheme 1:**
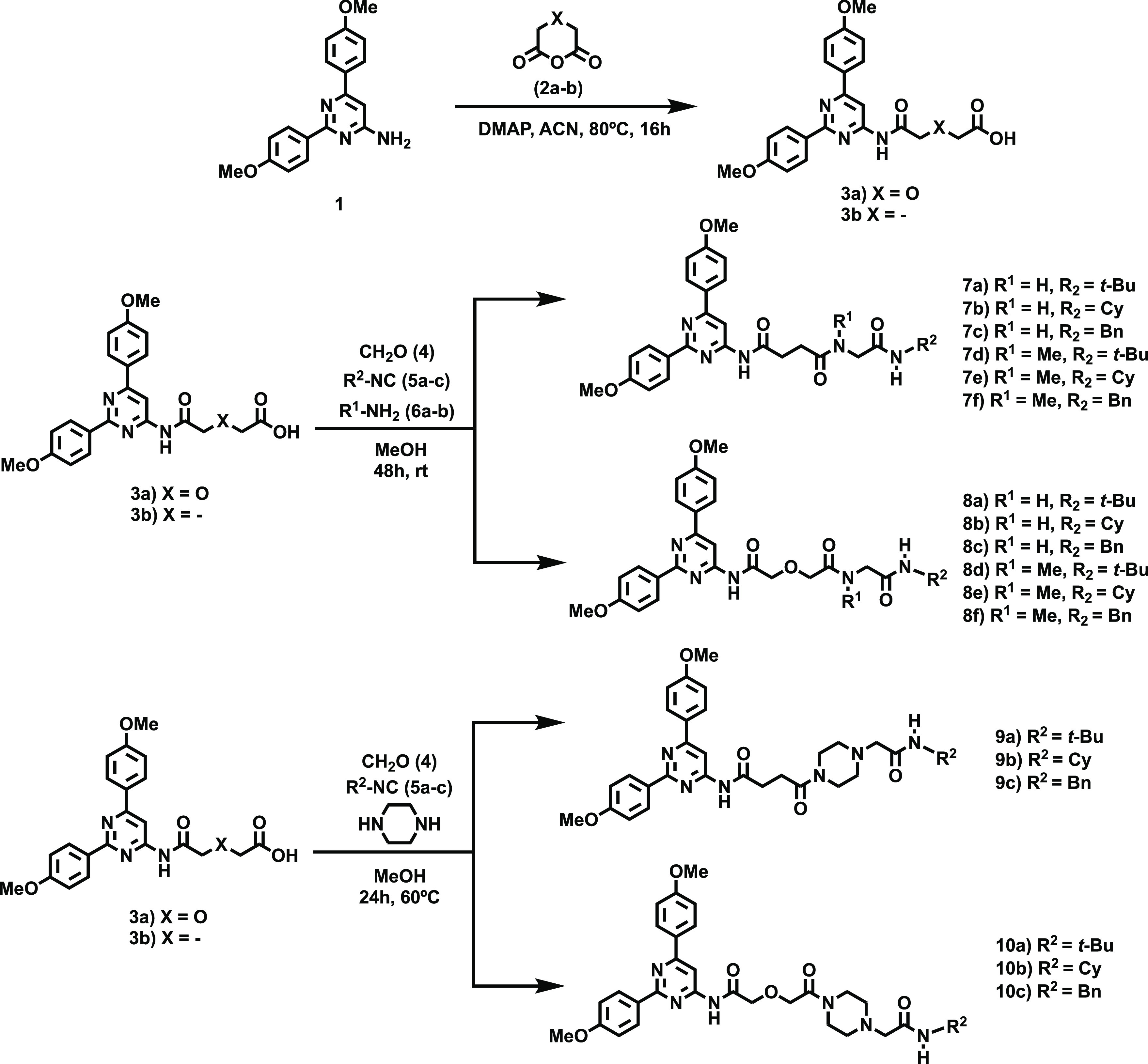
Ugi-Based Assembly of Designed 2-Amino-5-Substituted Pyrimidine Ligands (**7**–**10**)

The adenosinergic profile (affinity and selectivity) of the 18 novel pyrimidine derivatives (**7**–**10**) was evaluated in vitro (*h*AR subtypes) using radioligand binding experiments (Tables 1 and 2). Briefly, *h*ARs were expressed in transfected CHO (A_1_AR), HeLa (A_2A_AR and A_3_AR), and HEK-293 (A_2B_AR) cells. (^3^H)-8-Cyclopentyl-1,3-dipropylxanthine ([^3^H]DPCPX) for A_1_AR and [^3^H]NECA for A_3_AR were used as radioligands in the assays. Data obtained are expressed as *K*_i_ (nM, *n* = 3) or as percentage inhibition of specific binding at 1 μM (*n* = 2, mean) for compounds that did not fully displace the specific binding of the radioligand. *K*_i_ values were calculated by fitting the data with nonlinear regression using Prism 2.1 software (GraphPad). As a complement of the pharmacological characterization of the herein reported ligands, its A_3_AR affinity and those of the three reference AR antagonists (XAC, ISVY-130,^18^ and MRS 1220) were evaluated using a fluorescence polarization (FP) screening method (Tables 1 and 2). CELT-228, a potent (*K*_i_ = 52.7 nM) and highly selective commercially available A_3_AR fluorescent ligand (Celtarys Research), was employed. For calculating the affinity of the new compounds for the receptor, different concentrations of the test compounds were then incubated (30 min) at ambient temperature with membranes expressing human A_3_ receptors in 96-well plates in the presence of 75 nM CELT-228 and the fluorescence polarization was measured in each well. Representative curves obtained for selected compounds (**7d**, **9a**, and **10a**) are shown in Figure 2.

**Figure 2:**
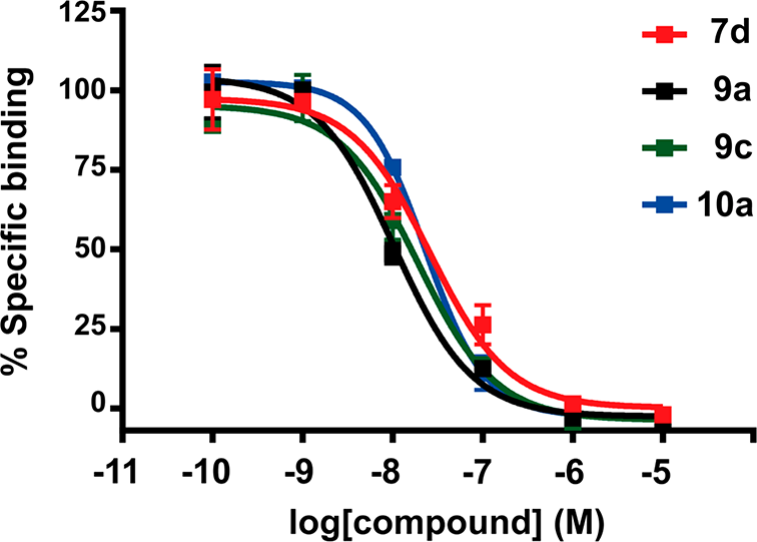
Concentration–percent of specific binding of CELT-228 curves obtained with compounds **7d** (red), **9a** (black), **9c** (green), and **10a** (blue). Points represent the mean ± SEM (vertical bars) of triplicate measurements.

**Table 1:**
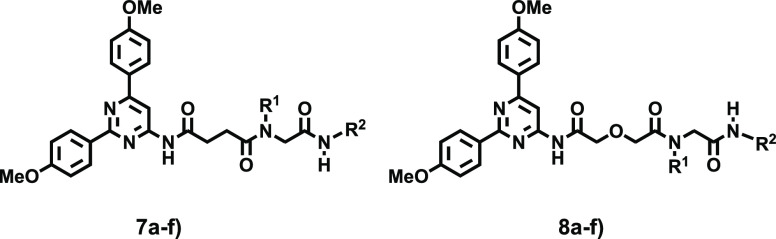
Structure and Affinity Binding Data for Series **I** (Ligands **7a**–**f** and **8a**–**f**) at the Human ARs

			*K*_i_ (nM) or % at 1 μM				
compd	R^1^	R^2^	*h*A_1_^a^	*h*A_2A_^b^	*h*A_2B_^c^	*h*A_3_^d^	*h*A_3_^e^
**7a**	H	*t*-Bu	10%	3%	4%	36.7 ± 7.2	15.0 ± 2.6
**7b**	H	Cy	5%	9%	16%	74.2 ± 10.9	35.1 ± 5.0
**7c**	H	Bn	2%	3%	3%	50.8 ± 9.0	26.9 ± 2.8
**7d**, ISAM-DM10	Me	*t*-Bu	32%	3%	10%	15.8 ± 4.3	4.6 ± 1.6
**7e**	Me	Cy	9%	11%	1%	52.9 ± 11.8	18.5 ± 4.5
**7f**	Me	Bn	32%	20%	2%	35.4 ± 11.2	9.5 ± 3.1
**8a**	H	*t*-Bu	7%	9%	3%	19.7 ± 2.7	11.2 ± 3.7
**8b**	H	Cy	6%	9%	3%	21.4 ± 1.5	14.0 ± 2.9
**8c**	H	Bn	9%	1%	2%	185.2 ± 20.7	64.6 ± 4.6
**8d**	Me	*t*-Bu	20%	4%	2%	19.4 ± 3.7	7.2 ± 1.1
**8e**	Me	Cy	13%	8%	2%	23.7 ± 2.2	10.1 ± 3.1
**8f**	Me	Bn	6%	1%	2%	40.1 ± 5.7	19.0 ± 3.7
XAC			29.1 ± 7.7	1.0 ± 0.2	141.0 ± 26.6	91.9 ± 16.1	25.3 ± 6.9
ISVY-130^18^			1%	10%	4%	3.60 ± 0.8	1.7 ± 0.6
MRS 1220						1.70 ± 0.1	1.4 ± 0.4

a Displacement of specific [^3^H]DPCPX binding in human CHO cells expressed as *K*_i_ ± SEM in nM (*n* = 3) or percentage displacement of specific binding at a concentration of 1 μM (*n* = 2).

b Displacement of specific [^3^H]4-(2-[7-amino-2-(2-furyl)[1,2,4]triazolo[2,3-*a*][1,3,5]triazin-5-ylamino]ethyl)phenol binding in human HeLa cells expressed as *K*_i_ ± SEM in nM (*n* = 3) or percentage displacement of specific binding at a concentration of 1 μM (*n* = 2).

c Displacement of specific [^3^H]DPCPX binding in human HEK-293 cells expressed as *K*_i_ ± SEM in nM (*n* = 3) or percentage displacement of specific binding at a concentration of 1 μM (*n* = 2).

d Displacement of specific [^3^H]NECA binding in human HeLa cells expressed as *K*_i_ ± SEM in nM (*n* = 3) or percentage displacement of specific binding at a concentration of 1 μM (*n* = 2).

e Displacement of specific binding of CELT-228 detected by means of fluorescence polarization measurements (*n* = 3). XAC (*N*-(2-aminoethyl)-2-(4-(2,6-dioxo-1,3-dipropyl-2,3,6,7-tetrahydro-1*H*-purin-8-yl)phenoxy)acetamide), ISVY-130 (*N*-(2,6-bis(4-methoxyphenyl)pyrimidin-4-yl)acetamide), and MRS 1220 (9-chloro-2-(2-furanyl)-5-((phenylacetyl)amino)-[1,2,4]triazolo[1,5-*c*]quinazoline) pharmacological data added as standard of A_3_AR antagonists.

**Table 2:**
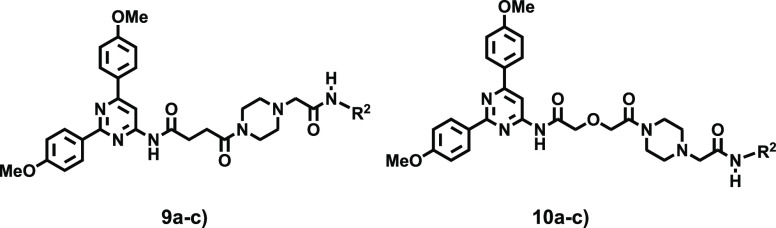
Structure and Affinity Binding Data for Series **II** (Ligands **9a**–**c** and **10a**–**c**) at the Human ARs

		*K*_i_ (nM) or % at 1 μM				
compd	R^2^	*h*A_1_^a^	*h*A_2A_^b^	*h*A_2B_^c^	*h*A_3_^d^	*h*A_3_^e^
**9a**, ISAM-DM13	*t*-Bu	19%	4%	1%	5.8 ± 0.7	1.8 ± 0.6
**9b**	Cy	9%	1%	1%	35.1 ± 6.4	10.5 ± 2.8
**9c**	Bn	6%	9%	2%	11.6 ± 3.3	3.4 ± 0.7
**10a**, ISAM-DM21	*t*-Bu	9%	36%	3%	13.6 ± 1.3	4.0 ± 1.2
**10b**	Cy	11%	3%	2%	16.1 ± 3.8	18.3 ± 2.6
**10c**	Bn	2%	7%	1%	33.0 ± 5.4	9.3 ± 3.1
XAC		29.1 ± 7.7	1.0 ± 0.2	141.0 ± 26.6	91.9 ± 16.1	25.3 ± 6.9
ISVY-130^18^		1%	10%	4%	3.60 ± 0.8	1.7 ± 0.6
MRS 1220					1.70 ± 0.1	1.4 ± 0.4

a Displacement of specific [^3^H]DPCPX binding in human CHO cells expressed as *K*_i_ ± SEM in nM (*n* = 3) or percentage displacement of specific binding at a concentration of 1 μM (*n* = 2).

b Displacement of specific [^3^H]4-(2-[7-amino-2-(2-furyl)[1,2,4]triazolo[2,3-*a*][1,3,5]triazin-5-ylamino]ethyl)phenol binding in human HeLa cells expressed as *K*_i_ ± SEM in nM (*n* = 3) or percentage displacement of specific binding at a concentration of 1 μM (*n* = 2).

c Displacement of specific [^3^H]DPCPX binding in human HEK-293 cells expressed as *K*_i_ ± SEM in nM (*n* = 3) or percentage displacement of specific binding at a concentration of 1 μM (*n* = 2).

d Displacement of specific [^3^H]NECA binding in human HeLa cells expressed as *K*_i_ ± SEM in nM (*n* = 3) or percentage displacement of specific binding at a concentration of 1 μM (*n* = 2).

e Displacement of specific binding of CELT-228 detected by means of fluorescence polarization measurements (*n* = 3). XAC (*N*-(2-aminoethyl)-2-(4-(2,6-dioxo-1,3-dipropyl-2,3,6,7-tetrahydro-1*H*-purin-8-yl)phenoxy)acetamide), ISVY-130 (*N*-(2,6-bis(4-methoxyphenyl)pyrimidin-4-yl)acetamide), and MRS 1220 (9-Chloro-2-(2-furanyl)-5-((phenylacetyl)amino)-[1,2,4]triazolo[1,5-*c*]quinazoline) pharmacological data added as standard of A_3_AR antagonists.

Tables 1 and 2 contain the binding data on the four ARs of series **I** and **II**, together with three reference AR antagonists (XAC, ISVY-130,^18^ and MRS 1220) measured under the same conditions. The potential promiscuity on a pan-assay interference compounds (PAINS) was ruled out on the whole set of ligands by in silico evaluation using the PAINS filter in RDkit^21^ With the aim to preliminarily validate the performance of a fluorescence polarization (FP) screening method, the A_3_AR binding affinity of the new ligands was evaluated by using both classical radioligand-based screening ([^3^H]NECA) and a FP assay (CELT-228). A comparative analysis of the binding data obtained (Tables 1 and 2) reveals that both assays provided affinity values that are in good agreement, although the fluorescence polarization assays provided slightly superior (not statistically significant) *K*_i_ values (mean *K*_i_ FP/radioligand ratio = 2.6 ± 0.8). These results support the use of fluorescence-based screening methods as alternatives to canonical binding assays (i.e., radioligand binding) with similar performance.

Examination of the reported *K*_i_ values (Tables 1 and 2) reveal the identification of several highly potent (*K*_i_ < 20 nM) A_3_AR ligands (e.g., **7d**, **8a**, **8d**, **9a**, **9c**, **10a**, and **10b**) that exhibit outstanding selectivity profile. The A_3_AR p*K*_*i*_ values are presented in Figure 3 to allow for a closer and effective analysis of SAR trends within the different subsets. A first observation is that, in both series, the *tert*-butyl group is the best residue at position R^2^, followed by cyclohexyl (Cy), regardless the nature of the linker or the substituent at R^1^ (Figure 3). Collectively, piperazine derivatives (series **II**) elicit superior A_3_AR affinity as compared to series **I**. More precisely, one can observe a tendency of affinities where piperazine > R^1^=N–Me > N^1^=NH. While the oxygenated linker (CH_2_–O–CH_2_) generally provided highly potent ligands in each subset (Figure 3), the effect of the linker nature on A_3_AR affinity seems to be nonsignificant. Interestingly, the most attractive A_3_AR antagonist discovered in the context of the study (**9a**, *h*A_3_AR *K*_i_ = 5.8 nM) derives of the ethyl liker.

**Figure 3:**
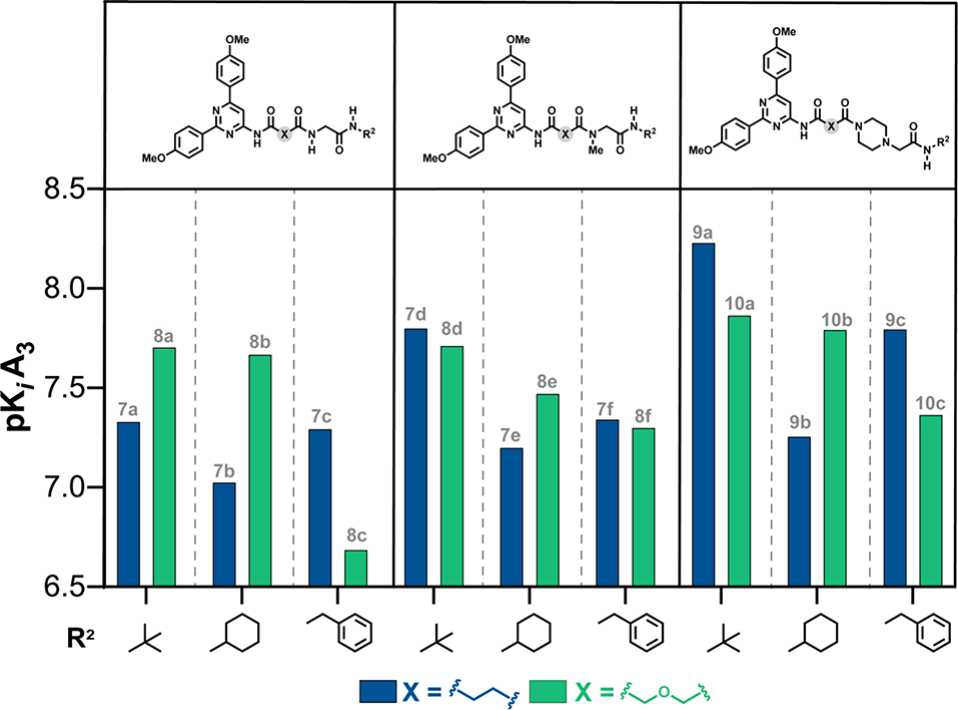
Comparative analysis of the SAR trends within the two subsets of ligands.

The SAR observations were then put in the context of the structural model that supported the design of these series. Every compound was independently docked by structural alignment of the central 2,6-diarylpyrimidines with ISVY-130, a 4-amido-2,6-diarylpyrimidine in complex with the A_3_AR homology model generated in our previous work, by means of homology modeling employing the A_2A_AR inactive structure (PDB: 3EML) as a template.^18^ This initial docking was followed by manual adjustment of the 4-amido substitution (L1), using the tools within the Maestro Schrödinger suite.^22^ In cases where more than one, significantly different alternative orientation for the flexible substituent at L1 was possible, every conformation was retained for the next stage, consisting of membrane insertion within a hexagonal-prism shaped box, containing a POPC bilayer patched water molecules, followed by a 5 ns molecular dynamics (MD) equilibration as implemented in the PyMemDyn routine within the GPCR-ModSim server.^23^ For each ligand, only the conformation with lowest ligand-RMSD and the highest number of protein–ligand interactions survived this stage (coordinates of each complex are provided in the Supporting Information). Finally, each equilibrated complex was subject to 3 × 100 ns unrestrained MD simulations with GROMACS,^24^ and used to assess the ligand stability and compute average number of protein–ligand polar interactions.

The results of the computational analysis are summarized on Figure 4. Here, one can appreciate that all molecules adopt a conserved binding mode for the orthosteric pharmacophore, governed by interactions with the conserved Asn250^6.55^ (double H-bond with the N^1^ and the exocyclic nitrogen of the N^4^-amido group; see Figure 4B) and a π–π stacking interaction with Phe168^EL2^. The variable substituent in L1 extends toward the extracellular vestibule, exploring additional non-orthosteric interactions with the receptor. Most ligands remained quite stable from the initial docking pose, and at least two out of the three MD replica simulations reached convergence (attending to the ligand RMSD) and were further analyzed. The region explored by the L1 elongation of the 4-amido-2,6-diarylpyrimidine is located at the interface between EL2 and EL3 and the tip of TM7 and TM2, with occasional interactions of the amide with Gln167^EL2^ (Figure 4). The positively charged nitrogen of the piperazine (series **II**) formed frequent salt-bridge interactions with Glu258^EL3^. As for the comparison between ligands with aliphatic linker (series **7**) or with the oxygenated linker (CH_2_–O–CH_2_, series **8**), we frequently observed that the ether induces additional flexibility resulting in a tendency of the substituent to bend over the orthosteric site, rather than extending toward the extracellular vestibule as in series **7**. In this model, no significant differences were observed between NH (**7d**–**f**) and methylated amide series (**8c**–**f**).

**Figure 4:**
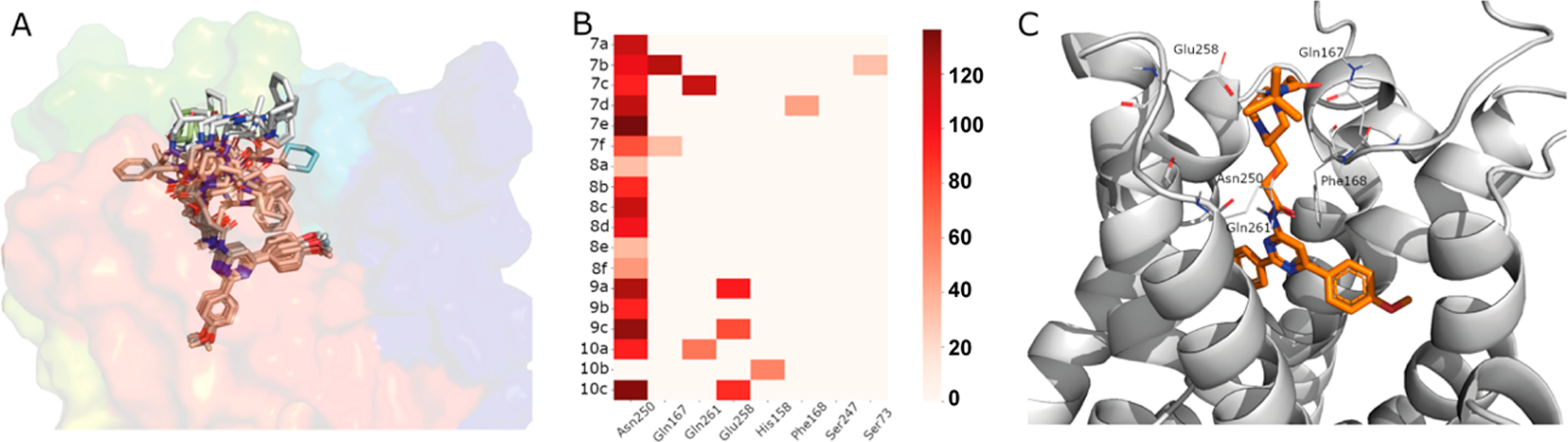
(A) Overlay of the representative pose for each compound, after docking to the hA_3_AR followed by MD simulation. (B) Heat map showing the H-bond occupancy (right-hand scale, in %, with values over 100% indicating a double H-bond, i.e., for Asn250, and a minimum cutoff of 30%) of the residues surrounding the L1 site (columns) for each ligand (rows). (C) Detailed view of compound **9a** in complex with the hA_3_AR, showing the residues participating on receptor–ligand contacts depicted in panel B.

In summary, we herein documented a focused library of pyrimidine-based compounds, functionalized on the L1 region, which retain the potent and highly selective profile of the original, orthosteric pharmacophore while improving the solubility. The molecular design aimed to explore non-orthosteric interactions within the A_3_AR, based on a binding model of the orthosteric scaffold to a homology-based model of the A_3_AR and further explored by MD simulations of the designed compound series. The structural functionalization could be performed in a rapid and efficient manner by using two Ugi-based approaches, and the pharmacological profiling on the AR family demonstrated the tolerability of the L1 region of the original, orthosteric pharmacophore for such structural functionalization. Additional studies are now underway to exploit the findings herein for the development of covalent drugs and molecular probes for diagnostic and delivery purposes.

## Supporting Information Available

Detailed experimental information: synthesis of target compounds, spectroscopic and analytical data, pharmacological binding assays and HPLC traces of best compounds (PDF)
3D coordinates of compounds and A_3_AR model (ZIP)

## Abbreviations

A_1_AR: A_1_ adenosine receptor
A_2A_AR: A_2A_ adenosine receptor
A_2B_AR: A_2B_ adenosine receptor
A_3_AR: A_3_ adenosine receptor
Ado: adenosine
AR: adenosine receptor
Cmpd: compound
Cy: cyclohexyl
MD: molecular dynamics
PAINS: pan-assay interference compounds
U-4CR: Ugi four component reaction.
