# Supplementary material for: Exploring Non-orthosteric Interactions with a Series of Potent and Selective A3 Antagonists
Source: ACS Med Chem Lett. 2022 Jan 10;13(2):243–9. doi: 10.1021/acsmedchemlett.1c00598 (PMC8842279; doi:10.1021/acsmedchemlett.1c00598)
Supplement: Supplementary file 1 — ml1c00598_si_001.pdf [file ml1c00598_si_001.pdf]

Exploring Non-Orthosteric Interactions with a Series of Potent and Selective A_3 Antagonists

Darío Miranda-Pastoriza,[1,2] Rodrigo Bernardez,[1,2] Jhonny Azuaje,[1,2] Rubén Prieto-Díaz,[1,2,3] Maria Majellaro, [1,2] Ashish V. Tamhankar,[3] Lucien Koenekoop,[3] Alejandro González,[4] Claudia Gioé-Gallo,[1,2] Ana Mallo-Abreu,[1,2] José Brea,[4*] M. Isabel Loza,[4] Aitor García-Rey, [1,2] Xerardo García-Mera,[2] Hugo Gutiérrez-de-Terán,[3*] and Eddy Sotelo[1,2*]

[1]Centro Singular de Investigación en Química Biolóxica e Materiais Moleculares (CIQUS) and [2]Departamento de Química Orgánica, Universidade de Santiago de Compostela, 15782 Santiago de Compostela, Spain. [3]Department of Cell and Molecular Biology, SciLifeLab, Uppsala University, Uppsala SE-75124. [4]Centro Singular de Investigación en Medicina Molecular y Enfermedades Crónicas (CIMUS). Universidade de Santiago de Compostela, 15782. Santiago de Compostela, Spain.

TABLE OF CONTENTS

Chemistry. General information..S2

General pathways for synthesis and spectroscopic and analytical data for compounds described...S3

Pharmacology. Binding assays..S11

HPLC traces of best compounds..S12

References...S15

Chemistry. General information

Commercially available starting materials, reagents and solvents were purchased and used without further purification. The reactions were monitored by thin-layer chromatography (TLC) with 2.5 mm Merck silica gel GF 254 strips, and the purified compounds each showed a single spot; unless stated otherwise, UV light, *p*-anisaldehyde solution and/or iodine vapor were used for detection of compounds. The Ugi reactions were performed in coated Kimble vials on a PLS (6X4) Organic Synthesizer with orbital stirring. Purity and identity of all tested compounds were established by a combination of mass spectrometry, HRMS and NMR spectra as described below. Purification of isolated products was carried out by column chromatography (Kieselgel 0.040–0.063 mm, E. Merck) or medium pressure liquid chromatography (MPLC) on a ComBiFlash Companion (Teledyne ISCO) with RediSep pre-packed normal-phase silica gel (35-60 µm) columns. Melting points were determined on a Stuart Scientific melting point apparatus and are uncorrected.

The NMR spectra were recorded on Bruker AM300 and XM500 spectrometers. Chemical shifts are given as δ values against tetramethylsilane as internal standard and J values are given in Hz. Mass spectra were obtained on a Varian MAT-711 instrument. High-resolution mass spectra were obtained on an Autospec Micromass spectrometer. The purity of all tested compounds was determined to be >95%. A detailed description of synthetic methodologies as well as analytical and spectroscopic data for all described compounds is described below.

Synthesis, spectroscopic and analytical data of compounds described:

General procedure for the synthesis of 2-(2-((2,6-bis(4-methoxyphenyl)pyrimidin-4-yl)amino)-2-oxoethoxy)acetic acid (3a) and 4-((2,6-bis(4-methoxyphenyl)pyrimidin-4-yl)amino)-4-oxobutanoic acid (3b).

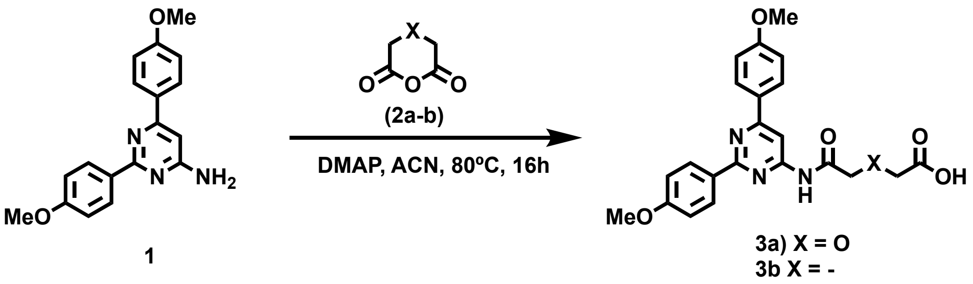

A mixture of 2,6-bis(4-methoxyphenyl)pyrimidin-4-amine[1] **1** (1 equiv.), diglycolic **2a** or succinic anhydride **2b** (1.5 equiv.) and 4-dimethylaminopyrimidine (DMAP, 0.1 equiv.) in acetonitrile (ACN, 5 mL) was stirred at 80ºC for 16h. After completion of the reaction, the solvent was evaporated under vacuum to afford an oily residue that was purified by chromatographic methods on silica gel using CH_2Cl_2/MeOH mixture.

2-(2-((2,6-bis(4-methoxyphenyl)pyrimidin-4-yl)amino)-2-oxoethoxy)acetic acid (3a). Yield: 61%. Mp. 195 – 197 ºC. ^{1}H NMR (300 MHz, DMSO-d_6) δ (ppm): 12.86 (s, 1H), 10.56 (s, 1H), 8.46 – 8.36 (m, 2H), 8.32 (s, 1H), 8.20 – 8.11 (m, 2H), 7.17 – 7.04 (m, 4H), 4.33 (s, 2H), 4.23 (s, 2H), 3.84 (s, 6H). MS (CI) *m/z* (%): 424.1 ([M+H]$^+$, 100).

4-((2,6-bis(4-methoxyphenyl)pyrimidin-4-yl)amino)-4-oxobutanoic acid (3b). Yield: 58%. Mp. 205 – 207 ºC. ^{1}H NMR (300 MHz, DMSO-d_6) δ (ppm): 12.19 (brs, 1H), 10.93 (brs, 1H), 8.49 – 8.39 (m, 2H), 8.36 (s, 1H), 8.21 – 8.12 (m, 2H), 7.17 – 7.06 (m, 4H), 3.86 (s, 6H), 2.75 (t, J = 6.6 Hz, 2H), 2.60 – 2.53 (m, 2H). MS (CI) *m/z* (%): 408.1 ([M+H]$^+$, 100).

General procedure for the synthesis of N_1-(2,6-bis(4-methoxyphenyl)pyrimidin-4-yl)-N_4-(2-(alkylamino)-2-oxoethyl)succinamides (7a-f) and N-(2,6-bis(4-methoxyphenyl)pyrimidin-4-yl)-2-(2-((2-(alkylamino)-2-oxoethyl)amino)-2-oxoethoxy)acetamides (8a-f)

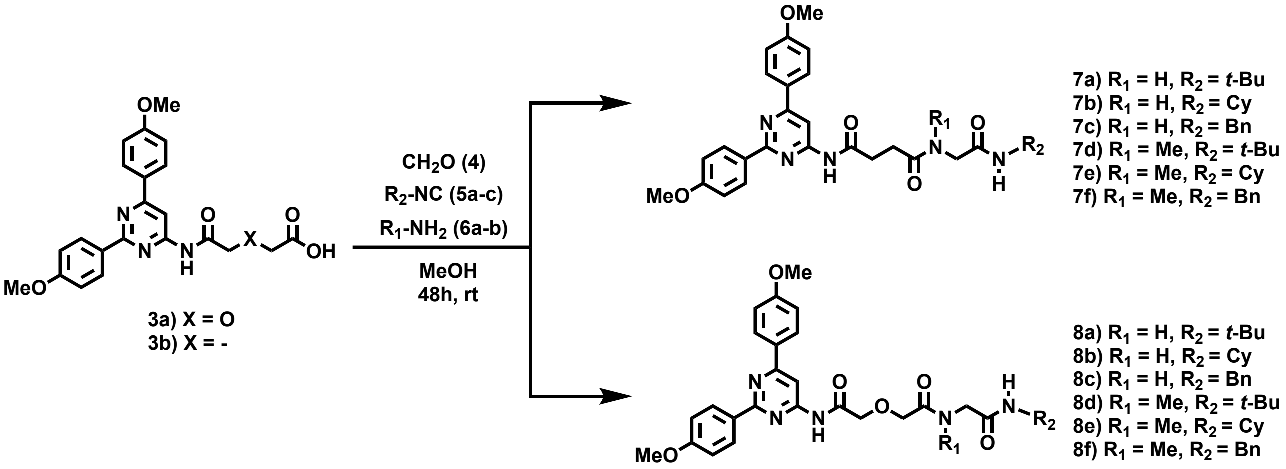

A mixture of the synthetised acid **3a-b** (1 equiv.), formaldehyde **4** (1 equiv.), the isocyanide **5a-c** (1 equiv.) and the amine **6a-b** (1 equiv) in MeOH (3 mL) was submitted to orbital stirring at room temperature for 48h. After completion of the reaction, CH_2Cl_2 (3 mL) and PS-*p*-TsOH (2 equiv.) were added. The reaction mixture was submitted to orbital stirring at room temperature until complete consumption of unreacted isocyanide (30-60 min). The polymeric reagent was filtered off and successively washed [2 times (4 mL)] with MeOH, AcOEt and CH_2Cl_2. Evaporation of the solvents from the filtrate afforded a residue, treated with water and extracted with ethyl acetate [3 times (15 mL)]. The organic phase was dried (Na_2SO_4) and evaporated under reduced pressure to afford an oily residue that was purified by chromatographic methods on silica gel using hexane/AcOEt mixtures.

N_1-(2,6-bis(4-methoxyphenyl)pyrimidin-4-yl)-N_4-(2-(tert-butylamino)-2-oxoethyl)succinamide (7a). Yield: 18%. Mp. 176 – 178 ºC. ^1H NMR (300 MHz, $CDCl_3$) δ (ppm): 8.44 (d, *J* = 8.5 Hz, 3H), 8.33 (s, 1H), 8.18 (dd, *J* = 9.1, 3.5 Hz, 2H), 7.00 (dd, *J* = 8.5, 2.1 Hz, 4H), 3.88 (s, 6H), 3.43 – 3.35 (m, 2H), 2.90 (s, 2H), 2.73 (s, 2H), 1.29 (s, 9H). MS (CI) *m/z* (%): 520.2 ($[M+H]^+$, 83).

N_1-(2,6-bis(4-methoxyphenyl)pyrimidin-4-yl)-N_4-(2-(cyclohexylamino)-2-oxoethyl)succinamide (7b). Yield: 19%. Mp. 202 – 204 ºC. ^1H NMR (300 MHz, $CDCl_3$) δ (ppm): 8.65 (s, 1H), 8.44 (dd, *J* = 8.9, 1.9 Hz, 2H), 8.32 (d, *J* = 3.3 Hz, 1H), 8.22 – 8.09 (m, 2H), 7.00 (ddd, *J* = 9.0, 4.2, 2.0 Hz, 4H), 3.88 (d, *J* = 2.1 Hz, 6H), 3.80 – 3.58 (m, 1H), 3.38 (d, *J* = 3.1 Hz, 2H), 2.88 (d, *J* = 11.1 Hz, 2H), 2.71 (t, *J* = 5.7 Hz, 2H), 2.05 – 0.77 (m, 10H). ^{13}C NMR (75 MHz, $CDCl_3$) δ (ppm): 171.8, 170.6, 170.2, 165.6, 164.2, 162.2, 160.2, 159.1, 133.7, 130.6, 129.8, 128.9, 114.0, 113.0, 102.0, 55.3, 48.5, 43.7, 32.5, 31.4, 31.0, 25.3, 24.6. MS (CI) *m/z* (%): 546.2 ($[M+H]^+$, 98).

N_1-(2-(benzylamino)-2-oxoethyl)-N_4-(2,6-bis(4-methoxyphenyl)pyrimidin-4-yl)succinamide (7c). Yield: 15%. Mp. 184 – 186 ºC. ^1H NMR (300 MHz, $CDCl_3$) δ (ppm): 8.65 – 8.31 (m, 3H), 8.31 – 8.00 (m, 2H), 7.35 – 7.07 (m, 5H), 7.07 – 6.84 (m, 5H), 4.40 (d, *J* = 5.9 Hz, 2H), 3.87 (d, *J* = 2.4 Hz, 6H), 3.34 (s, 2H), 2.98 – 2.62 (m, 4H). MS (CI) *m/z* (%): 554.2 ($[M+H]^+$, 75).

N_1-(2,6-bis(4-methoxyphenyl)pyrimidin-4-yl)-N_4-(2-(tert-butylamino)-2-oxoethyl)-N_4-methylsuccinamide (7d). Yield: 22%. Mp. 136 – 138 ºC. ^1H NMR (300 MHz, DMSO-d_6) δ (ppm): 10.87 (d, *J* = 1.9 Hz, 1H), 8.49 – 8.35 (m, 2H), 8.34 (d, *J* = 2.1 Hz, 1H), 8.24 – 7.96 (m, 2H), 7.62 (s, 1H), 7.17 – 7.00 (m, 4H), 3.91 (s, 2H), 3.84 (s, 6H), 3.33 – 3.22 (m, 3H), 2.99 (s, 2H), 2.76 (s, 2H), 1.25 (d, *J* = 15.8 Hz, 9H). ^{13}C NMR (75 MHz, DMSO-d_6) δ (ppm): 173.7, 172.3, 171.9, 167.9, 164.3, 163.3, 162.0, 159.5, 130.3, 129.9, 129.7, 128.9, 114.8, 114.3, 101.4, 55.8, 55.8, 52.8, 50.9, 36.4, 36.4, 34.8, 34.8, 31.9, 29.0. HRMS (APCI) *m/z* calcd for $C_{29}H_{36}N_5O_5$ $[M+H]^+$: 534.2711; found: 534.2719.

N_1-(2,6-bis(4-methoxyphenyl)pyrimidin-4-yl)-N_4-(2-(cyclohexylamino)-2-oxoethyl)-N_4-methyl-succinamide (7e). Yield: 21% Mp. 223 – 225 ºC. ^{1}H NMR (300 MHz, $CDCl_3$) δ (ppm): 8.58 – 8.39 (m, 2H), 8.35 (s, 1H), 8.19 (d, J = 8.4 Hz, 2H), 7.00 (d, J = 8.4 Hz, 4H), 6.14 (s, 1H), 4.03 (s, 2H), 3.89 (d, J = 1.5 Hz, 6H), 3.19 (s, 3H), 3.11 – 2.43 (m, 5H), 2.10 – 0.70 (m, 10H). MS (CI) *m/z* (%): 520.2 ($[M+H]^+$, 43).

N_1-(2-(benzylamino)-2-oxoethyl)-N_4-(2,6-bis(4-methoxyphenyl)pyrimidin-4-yl)-N_1-methylsucci-namide (7f). Yield: 31%. Mp. 220 – 222 ºC. ^{1}H NMR (300 MHz, DMSO-d_6) δ (ppm): 10.87 (s, 1H), 8.57 (s, 1H), 8.42 (d, J = 8.3 Hz, 2H), 8.31 (s, 1H), 8.12 (d, J = 8.3 Hz, 2H), 7.85 – 6.80 (m, 9H), 4.61 – 4.14 (m, 2H), 4.11 – 3.89 (m, 2H), 3.83 (s, 6H), 3.05 (s, 3H), 2.93 – 2.69 (m, 4H). MS (CI) *m/z* (%): 560.1 ($[M+H]^+$, 82).

N-(2,6-bis(4-methoxyphenyl)pyrimidin-4-yl)-2-(2-((2-(tert-butylamino)-2-oxoethyl)amino)-2-oxo-ethoxy)acetamide (8a). Yield: 23%. Oil. ^{1}H NMR (300 MHz, $CDCl_3$) δ (ppm): 9.60 (s, 1H), 8.50 (d, J = 8.3 Hz, 2H), 8.43 (s, 1H), 8.30 – 8.15 (m, 2H), 7.20 – 6.89 (m, 4H), 6.00 (s, 1H), 4.53 (s, 2H), 4.28 (s, 2H), 3.89 (s, 6H), 3.39 (s, 2H), 1.34 (s, 9H). ^{13}C NMR (75 MHz, $CDCl_3$) δ (ppm): 170.3, 168.8, 167.3, 165.3, 163.7, 161.9, 161.8, 157.6, 130.4, 129.9, 129.9, 129.0, 114.1, 113.7, 101.8, 71.6, 69.5, 55.4, 52.1, 51.9, 28.7. MS (CI) *m/z* (%): 534.2 ($[M+H]^+$, 100).

N-(2,6-bis(4-methoxyphenyl)pyrimidin-4-yl)-2-(2-((2-(cyclohexylamino)-2-oxoethyl)amino)-2-oxo-ethoxy)acetamide (8b). Yield: 36%. Mp. 113 – 115 ºC. ^{1}H NMR (300 MHz, $CDCl_3$) δ (ppm): 9.57 (s, 1H), 8.50 (d, J = 8.3 Hz, 2H), 8.42 (s, 1H), 8.22 (d, J = 8.7 Hz, 2H), 7.01 (dd, J = 8.8, 6.8 Hz, 4H), 6.19 (brs, J = 8.0 Hz, 1H), 4.53 (s, 2H), 4.27 (s, 2H), 3.88 (s, 6H), 3.81 – 3.55 (m, 1H), 3.39 (s, 2H), 2.21 – 0.92 (m, 10H). ^{13}C NMR (75 MHz, $CDCl_3$) δ (ppm): 170.7, 169.1, 167.5, 165.6, 162.2, 162.1, 157.9, 154.2, 130.7, 130.2, 129.3, 114.4, 114.0, 102.1, 71.9, 69.5, 56.4, 55.7, 51.8, 48.6, 33.1, 25.7, 24.9. MS (CI) *m/z* (%): 561.5 ($[M+H]^+$, 100).

N-benzyl-2-(2-(2-((2,6-bis(4-methoxyphenyl)pyrimidin-4-yl)amino)-2-oxoethoxy)acetamido) acetamide (8c). Yield: 25%. Mp. 201 – 203 ºC. ^{1}H NMR (300 MHz, $CDCl_3$) δ (ppm): 9.60 (s, 1H), 8.49 (d, *J* = 8.1 Hz, 2H), 8.39 (s, 1H), 8.23 (d, *J* = 8.5 Hz, 2H), 7.50 – 7.11 (m, 6H), 7.00 (dd, *J* = 8.5, 8.1 Hz, 4H), 4.48 (s, 2H), 4.40 (s, 2H), 4.24 (s, 2H), 4.07 (s, 2H), 3.88 (s, 6H). MS (CI) *m/z* (%): 570.5 ($[M+H]^+$, 73).

2-(2-((2,6-bis(4-methoxyphenyl)pyrimidin-4-yl)amino)-2-oxoethoxy)-N-(2-(tert-butylamino)-2-oxoethyl)-N-methylacetamide (8d). Yield: 35%. Mp. 115 – 117 ºC. ^{1}H NMR (300 MHz, $cdcl_3$) δ (ppm): 9.63 (s, 1H), 8.49 (d, *J* = 8.9 Hz, 2H), 8.42 (s, 1H), 8.22 (d, *J* = 8.8 Hz, 2H), 7.07 – 6.93 (m, 4H), 4.45 (s, 2H), 4.28 (s, 2H), 3.94 (d, *J* = 1.5 Hz, 2H), 3.88 (s, 6H), 3.07 (s, 3H), 1.35 (s, 9H). ^{13}C NMR (75 MHz, DMSO-d_6) δ (ppm): 170.8, 170.8, 170.3, 164.6, 163.4, 162.1, 162.1, 156.7, 130.1, 130.0, 129.5, 129.0, 114.9, 114.4, 101.5, 69.6, 67.1, 55.9, 55.8, 50.9, 34.8, 28.9. MS (CI) *m/z* (%): 550.2 ($[M+H]^+$, 100).

2-(2-((2,6-bis(4-methoxyphenyl)pyrimidin-4-yl)amino)-2-oxoethoxy)-N-(2-(cyclohexylamino)-2-oxoethyl)-N-methylacetamide (8e). Yield: 37%. Mp. 186 – 188 ºC. ^{1}H NMR (300 MHz, $cdcl_3$) δ (ppm): 9.69 (s, 1H), 8.50 (d, *J* = 8.5 Hz, 2H), 8.42 (s, 1H), 8.22 (d, *J* = 8.6 Hz, 2H), 7.00 (t, *J* = 8.1 Hz, 4H), 4.44 (s, 2H), 4.26 (s, 2H), 3.99 (s, 2H), 3.88 (s, 6H), 3.76 (s, 1H), 3.14 – 3.01 (m, 3H), 2.16 – 0.79 (m, 10H). ^{13}C NMR (75 MHz, $CDCl_3$) δ (ppm): 169.8, 169.3, 167.4, 165.6, 164.0, 162.2, 162.1, 157.9, 130.7, 130.3, 130.2, 129.3, 114.4, 114.0, 102.1, 72.0, 69.7, 55.7, 52.8, 48.7, 35.8, 33.2, 25.7, 25.0. MS (CI) *m/z* (%): 576.6 ($[M+H]^+$, 100).

N-(2-(benzylamino)-2-oxoethyl)-2-(2-((2,6-bis(4-methoxyphenyl)pyrimidin-4-yl)amino)-2-oxoethoxy)-N-methylacetamide (8f). Yield: 29%. Mp. 166 – 168 ºC. ^{1}H NMR (300 MHz, $CDCl_3$) δ (ppm): 9.68 (s, 1H), 8.62 – 8.43 (m, 2H), 8.40 (s, 1H), 8.31 – 8.13 (m, 2H), 7.41 – 7.15 (m, 5H), 7.10 – 6.89 (m, 4H), 6.76 – 6.45 (m, 1H), 4.55 – 4.27 (m, 4H), 4.22 (d, *J* = 7.7 Hz, 2H), 4.08 (s, 2H), 3.89 (s, 6H), 3.06 (s, 3H). MS (CI) *m/z* (%): 584.2 ($[M+H]^+$, 88).

General procedure for the synthesis of N-(2,6-bis(4-methoxyphenyl)pyrimidin-4-yl)-4-(4-(2-(alkylamino)-2-oxoethyl)piperazin-1-yl)-4-oxobutanamides (9a-c) and *N-(2,6-bis(4-methoxyphenyl)pyrimidin-4-yl)-2-(2-(4-(2-(alkylamino)-2-oxoethyl)piperazin-1-yl)-2-oxoethoxy)acetamides* (10a-c)

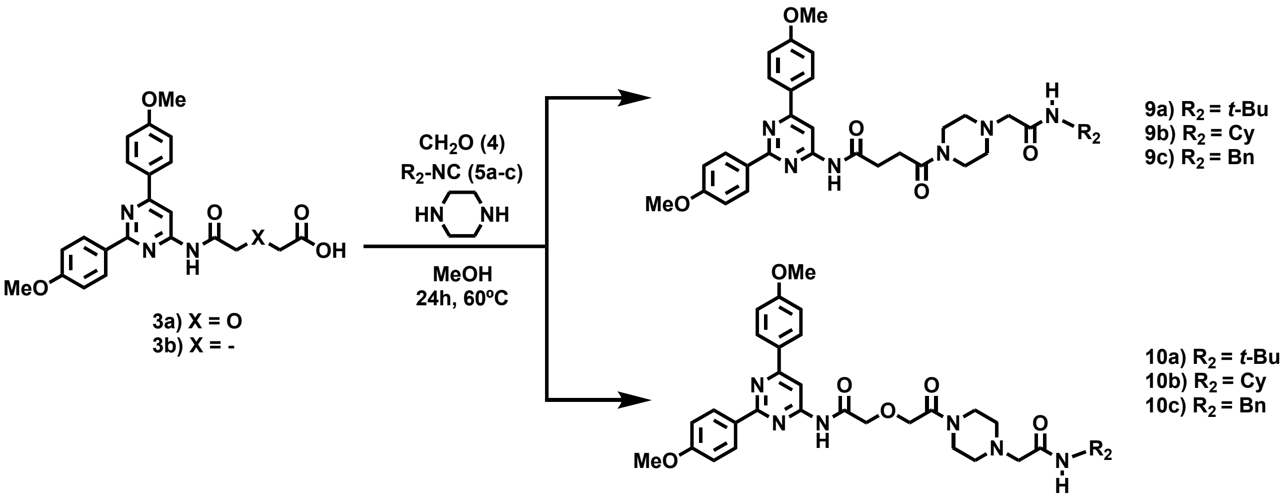

A mixture of the synthetised acid **3a-b** (1 equiv.), formaldehyde **4** (1 equiv.), the isocyanide **5a-c** (1 equiv.) and piperidine (1 equiv) in MeOH (3 mL) was submitted to orbital stirring at 60ºC for 24h. After completion of the reaction, CH_2Cl_2 (3 mL) and PS-*p*-TsOH (2 equiv.) were added. The reaction mixture was submitted to orbital stirring at room temperature until complete consumption of unreacted isocyanide (30-60 min). The polymeric reagent was filtered off and successively washed [2 times (4 mL)] with MeOH, AcOEt and CH_2Cl_2. Evaporation of the solvents from the filtrate afforded a residue, treated with water and extracted with ethyl acetate [3 times (15 mL)]. The organic phase was dried (Na_2SO_4) and evaporated under reduced pressure to afford an oily residue that was purified by chromatographic methods on silica gel using hexane/AcOEt mixtures.

N-(2,6-bis(4-methoxyphenyl)pyrimidin-4-yl)-4-(4-(2-(tert-butylamino)-2-oxoethyl)piperazin-1-yl)-4-oxobutanamide (9a). Yield: 27%. Mp. 203 – 205 ºC. ^{1}H NMR (300 MHz, $CDCl_3$) δ (ppm): 8.92 (s, 1H), 8.36 (s, 1H), 8.31 (s, 2H), 8.26 – 8.12 (m, 2H), 7.08 – 6.94 (m, 4H), 3.88 (t, *J* = 1.9 Hz, 6H), 3.68 (t, *J* = 5.1 Hz, 2H), 3.53 (t, *J* = 5.2 Hz, 2H), 3.03 – 2.70 (m, 6H), 2.51 (dt, *J* = 17.7, 4.9 Hz, 4H), 1.37 (d, *J* = 1.4 Hz, 9H). ^{13}C NMR (75 MHz, DMSO-d_6) δ (ppm): 173.6, 170.0, 168.7, 164.3, 163.3, 162.0, 162.0, 159.5, 130.3, 129.9, 129.7, 128.9, 114.9, 114.4, 101.4, 61.8, 55.8, 53.2, 50.3, 45.1, 31.9, 28.9, 27.7. HRMS (APCI) *m/z* calcd for $C_{32}H_{41}N_6O_6$ $[M+H]^+$: 589.3133; found: 589.3154.

N-(2,6-bis(4-methoxyphenyl)pyrimidin-4-yl)-4-(4-(2-(cyclohexylamino)-2-oxoethyl)piperazin-1-yl)-4-oxobutanamide (9b). Yield: 27%. Oil. ^{1}H NMR (300 MHz, $CDCl_3$) δ (ppm): 8.81 (d, *J* = 2.3 Hz, 1H), 8.50 – 8.39 (m, 2H), 8.36 (d, *J* = 1.9 Hz, 1H), 8.25 – 8.13 (m, 2H), 7.06 – 6.84 (m, 4H), 3.87 (s, 6H), 3.85 – 3.74 (m, 1H), 3.72 – 3.43 (m, 4H), 3.01 (d, *J* = 2.1 Hz, 2H), 2.92 – 2.71 (m, 2H), 2.71 – 2.43 (m, 6H), 1.89 (d, *J* = 12.2 Hz, 2H), 1.76 – 1.55 (m, 4H), 1.39 (q, *J* = 12.1 Hz, 2H), 1.30 – 1.07 (m, 2H). MS (CI) *m/z* (%): 615.7 ($[M+H]^+$, 72).

4-(4-(2-(benzylamino)-2-oxoethyl)piperazin-1-yl)-N-(2,6-bis(4-methoxyphenyl)pyrimidin-4-yl)-4-oxobutanamide (9c). Yield: 17%. Mp. 194 – 195 ºC. ^{1}H NMR (300 MHz, $CDCl_3$) δ (ppm): 8.67 (s, 1H), 8.53 – 8.40 (m, 2H), 8.37 (s, 1H), 8.21 (dd, *J* = 8.7, 1.8 Hz, 2H), 7.52 – 7.13 (m, 5H), 7.13 – 6.84 (m, 4H), 4.50 (d, *J* = 5.9 Hz, 2H), 3.90 (s, 6H), 3.76 – 3.39 (m, 4H), 3.25 – 3.10 (m, 2H), 2.90 – 2.69 (m, 4H), 2.61 (s, 4H). ^{13}C NMR (75 MHz, $CDCl_3$) δ (ppm): 172.3, 170.2, 168.6, 165.5, 162.2, 162.1, 158.7, 158.2, 138.6, 130.6, 130.3, 130.1, 129.3, 129.1, 128.2, 127.9, 114.4, 114.0, 101.9, 61.7, 55.7, 53.6, 43.4, 42.0, 32.9, 28.5. HRMS (APCI) *m/z* calcd for $C_{35}H_{39}N_6O_5$ $[M+H]^+$: 623.2976; found: 623.2985.

N-(2,6-bis(4-methoxyphenyl)pyrimidin-4-yl)-2-(2-(4-(2-(tert-butylamino)-2-oxoethyl)piperazin-1-yl)-2-oxoethoxy)acetamide (10a). Yield: 28%. Mp. 106 – 108 ºC. ^{1}H NMR (300 MHz, $CDCl_3$) δ (ppm): 99.64 (s, 1H), 8.54 – 8.42 (m, 2H), 8.39 (s, 1H), 8.27 – 8.14 (m, 2H), 6.98 (dd, *J* = 8.8, 6.7 Hz, 4H), 4.34 (s, 2H), 4.24 (s, 2H), 3.86 (s, 6H), 3.68 (q, *J* = 4.7 Hz, 2H), 3.38 (t, *J* = 4.9 Hz, 2H), 2.92 (s, 2H), 2.52 (t, *J* = 5.0 Hz, 4H), 1.38 – 1.30 (m, 9H). HRMS (APCI) *m/z* calcd for $C_{32}H_{41}N_6O_6$ $[M+H]^+$: 605.3082; found: 604.3095.

N-(2,6-bis(4-methoxyphenyl)pyrimidin-4-yl)-2-(2-(4-(2-(cyclohexylamino)-2-oxoethyl)piperazin-1-yl)-2-oxoethoxy)acetamide (10b). Yield: 17%. Oil. ^{1}H NMR (300 MHz, $CDCl_3$) δ (ppm): 9.61 (s, 1H), 8.48 (d, *J* = 8.6 Hz, 2H), 8.39 (s, 1H), 8.21 (d, *J* = 8.5 Hz, 2H), 6.99 (dd, *J* = 8.7, 6.8 Hz, 4H), 4.37 (s, 2H), 4.19 (s, 2H), 3.87 (s, 6H), 3.74 (s, 5H), 3.00 (d, *J* = 5.8 Hz, 2H), 2.67 – 2.37 (m, 4H), 2.26 – 0.20 (m, 10H). ^{13}C NMR (75 MHz, $CDCl_3$) δ (ppm): 168.8, 168.0, 166.9, 165.3, 163.7, 161.9, 161.8, 157.6, 130.4, 129.9, 129.0, 114.1, 113.7, 101.8, 71.6, 69.6, 61.5, 55.4, 55.4, 53.2, 47.5, 44.4, 41.9, 33.1, 33.0, 25.5, 24.7. HRMS (APCI) *m/z* calcd for $C_{34}H_{43}N_6O_6$ $[M+H]^+$: 631.3239; found: 631.3248.

N-benzyl-2-(4-(2-(2-((2,6-bis(4-methoxyphenyl)pyrimidin-4-yl)amino)-2-oxoethoxy)acetyl)piperazin-1-yl)acetamide (10c). Yield: 28%. Oil. ^{1}H NMR (300 MHz, $CDCl_3$) δ (ppm): 9.58 (s, 1H), 8.48 (d, *J* = 8.7 Hz, 2H), 8.40 (s, 1H), 8.22 (d, *J* = 8.9 Hz, 2H), 7.48 – 7.12 (m, 5H), 7.12 – 6.85 (m, 4H), 4.48 (s, 2H), 4.36 (s, 2H), 4.25 (s, 2H), 3.88 (s, 6H), 3.72 – 3.54 (m, 2H), 3.43 – 3.29 (m, 2H), 3.08 (d, *J* = 19.1 Hz, 2H), 2.69 – 2.42 (m, 4H). ^{13}C NMR (75 MHz, $CDCl_3$) δ (ppm): 169.2, 168.8, 166.9, 165.3, 163.7, 161.9, 161.8, 157.6, 138.2, 130.4, 129.9, 129.0, 128.8, 128.7, 127.6, 127.6, 127.5, 114.1, 113.7, 101.8, 71.6, 69.6, 61.5, 55.4, 55.4, 53.3, 53.1, 44.4, 43.1. MS (CI) *m/z* (%): 639.7 ($[M+H]^+$, 100).

Pharmacology. Binding assays

The adenosinergic profile (affinity and selectivity) of the 18 novel pyrimidine derivatives (**7-10**) was evaluated *in vitro* (four human AR subtypes) using radioligand binding assays. Human adenosine receptors were expressed in transfected CHO (A_1AR), HeLa (A_{2A}AR and A_3AR) and HEK-293 (A_{2B}AR) cells. (^{3}H)-1,3-Dipropyl-8-cyclopentylxanthine ([^{3}H]DPCPX) for A_1AR and [^{3}H]NECA for A_3AR were employed as radioligands in the assays. The biological data are expressed as K_i (nM, n = 3) or as percentage inhibition of specific binding at 1 µM (n = 2, average) for those compounds that did not fully displace specific radioligand binding. K_i values were obtained by fitting the data with nonlinear regression using Prism 2.1 software (GraphPad, San Diego, CA).

Furthermore, for A_3AR, fluorescence polarization binding experiments were carried out in a 96-well plate (Corning 3650). D-PBS (Sigma D8537) was used as assay buffer. In each well, 100µg of membranes from Hela-A3 cell line previously prepared in our laboratory (Lot: A006/ 17-01-2020, protein concentration= 3408 µg/ml) were preincubated for 10 minutes at RT along with compounds used as standard and non-specific binding determined in presence of R-PIA 10µM (Sigma S739). 75 nM of the fluorescent ligand (CELT-228) was added, and the reaction mixture (Vt = 200µl) was incubated for 30 minutes at RT. The polarized fluorescence signal was read in an Infinite M1000 pro multiplate reader (Tecan 30063849). For validation of fluorescence polarization binding assay MRS-1220 (*h*A_3AR K_i described: 1.7 nM) was used as a control, obtaining a *h*A_3AR K_i of 1.4 nM. The graphic below shows a representation of the concentration-response curves of the MRS-1220.

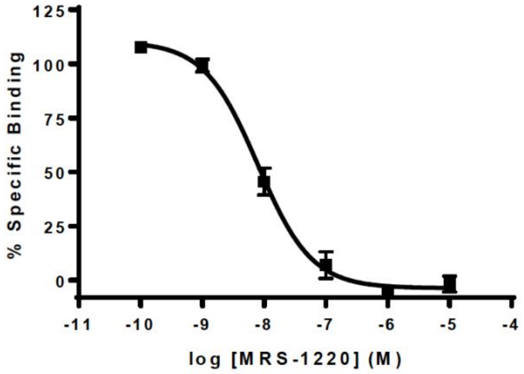

HPLC traces of best compounds

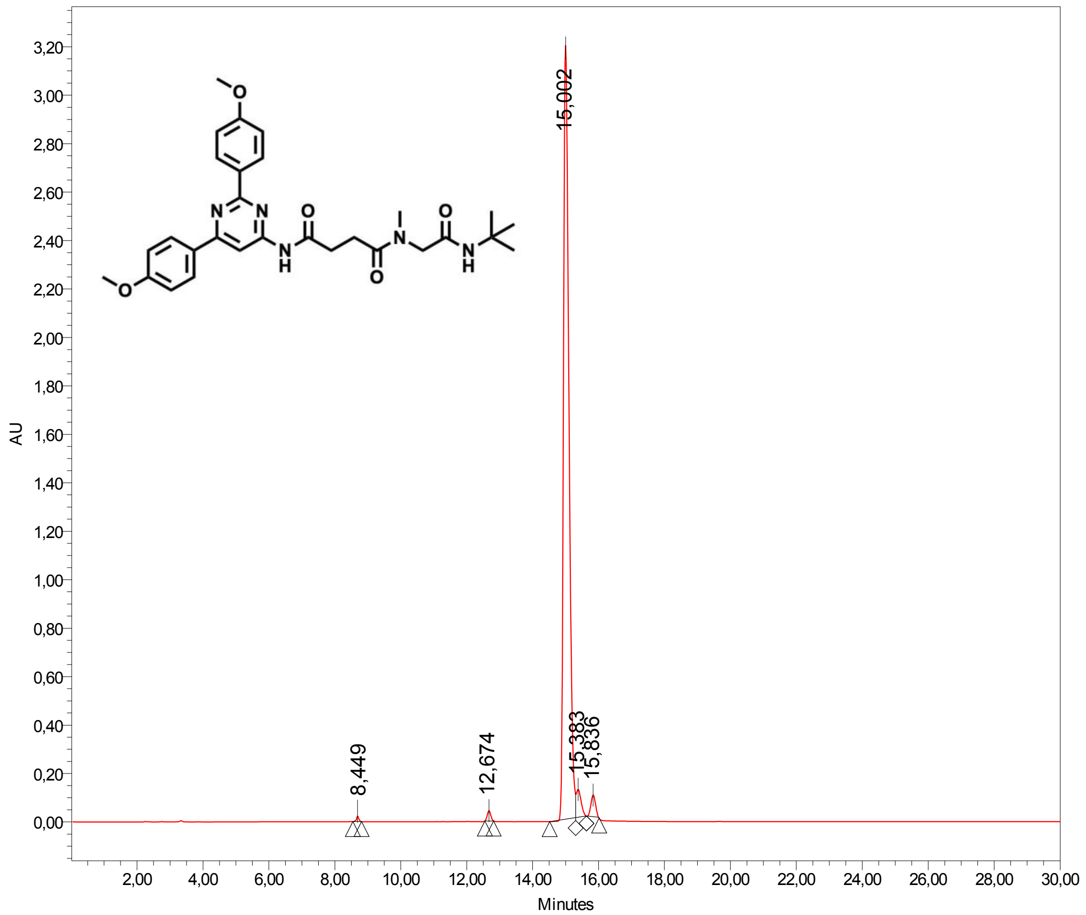

Sample Name: ISAM-DM10; Date Acquired: 23/07/2021 15:40:55 CET; Vial: 1; Injection: 1

Peak Summary with Statistics
Peak Name:

	Sample Name	Vial	Inj.	RT (min)	% Area
1	sy1dm-10	1	1	8,449	0,01
2	sy1dm-10	1	1	12,674	0,80
3	sy1dm-10	1	1	15,836	1,89
4	sy1dm-10	1	1	15,383	2,09
5	sy1dm-10	1	1	15,002	95,21

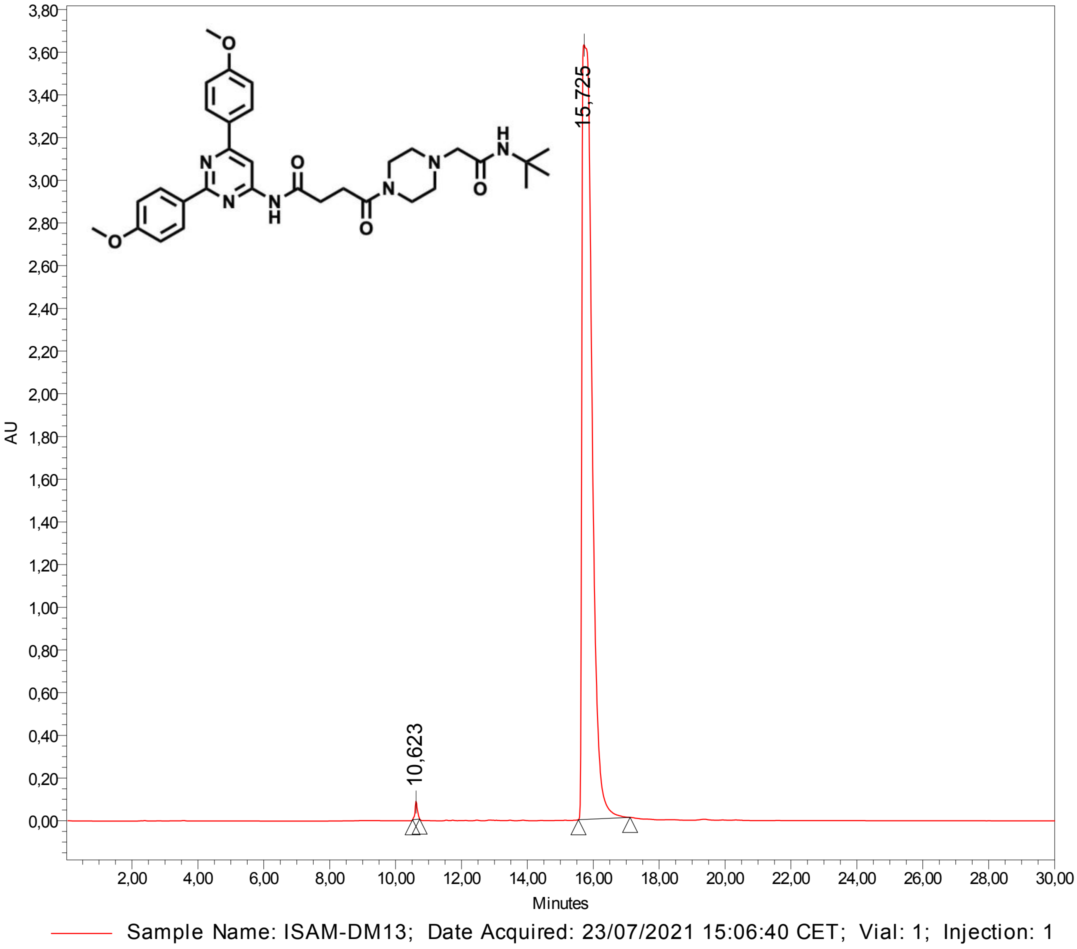

Peak Summary with Statistics
Peak Name:

	Sample Name	Vial	Inj.	RT (min)	Area (µV*sec)	% Area	Height (µV)
1	sy1dm-13	1	1	15,725	77348717	99,46	3634316
2	sy1dm-13	1	1	10,623	419588	0,54	82024

Instrument Method: 10%DCMiPAGradF1

Stored: 23/07/2021 11:26:08 CET

Method Information

Method Modified User	Breeze
Method Version	1
Method Edit User	

PCM/15xx Instrument Setup

Type	PCM/15xx	High Limit	4000,0	Solvent C
Instrument Status	On	Low Limit	0,0	
Pump Mode	Gradient	Total Flow	1,00	
Flow A	1,00	Use Events	Off	
Flow B	0,00	Solvent A	DCM	
Flow C	0,00	Solvent B	iPA	

PCM/15xx Gradient Table

	Time	Flow	%A	%B	%C	Curve
1		1,00	100,0	0,0	0,0	
2	30,00	1,00	90,0	10,0	0,0	6

W2487 Instrument Setup

Type	W2487
Instrument Status	On
Dual Wavelength	True
Pulse Period Seconds	1,0
Pulse Repeat Period Seconds	1,0

W2487 Channel Information

Channel Name	2487Channel 1	Voltage Offset	0
Description	254 nm	Polarity	+
Use Channels	On	AutoZero Wavelength	True
Wavelength	254	AutoZero Keypad	True
Output Mode	Absorbance A (Ch1)	AutoZeroEvent Input	True
Data Mode	Absorbance A (Ch1)	AutoZero Inject	True
Sampling Rate	1	Chart Mark Enable	True
Filter Type	Hamming	Ratio AuMinimum	0,1000
Aufs	2,0000	Minimum Ratio	0,00
Time Constant	1,0	Maximum Ratio	2,00
AU Offset	0,000		

W2487 Channel Information

Channel Name	2487Channel 2	Wavelength	280
Description	280 nm	Output Mode	Absorbance B (Ch2)
Use Channels	On	Data Mode	Absorbance B (Ch2)

References

(1) Yaziji, V.; Rodríguez, D.; Gutiérrez-De-Terán, H.; Coelho, A.; Caamaño, O.; García-Mera, X.; Brea, J.; Loza, M. I.; Cadavid, M. I.; Sotelo, E. Pyrimidine derivatives as potent and selective A_3 adenosine receptor antagonists. *J. Med. Chem.* **2011**, *54* (2), 457-471.
